# Proteomic analysis of proteome and histone post-translational modifications in heat shock protein 90 inhibition-mediated bladder cancer therapeutics

**DOI:** 10.1038/s41598-017-00143-6

**Published:** 2017-03-15

**Authors:** Qingdi Quentin Li, Jian-Jiang Hao, Zheng Zhang, L. Spencer Krane, Kai H. Hammerich, Thomas Sanford, Jane B. Trepel, Len Neckers, Piyush K. Agarwal

**Affiliations:** 1 Urologic Oncology Branch, Center for Cancer Research, National Cancer Institute, National Institutes of Health, Bethesda, Maryland 20892 USA; 2 Poochon Scientific, Frederick, Maryland 21701 USA; 3 Developmental Therapeutics Branch, Center for Cancer Research, National Cancer Institute, National Institutes of Health, Bethesda, Maryland 20892 USA

## Abstract

Heat shock protein 90 (HSP90) inhibition is an attractive strategy for cancer treatment. Several HSP90 inhibitors have shown promising effects in clinical oncology trials. However, little is known about HSP90 inhibition-mediated bladder cancer therapy. Here, we report a quantitative proteomic study that evaluates alterations in protein expression and histone post-translational modifications (PTMs) in bladder carcinoma in response to HSP90 inhibition. We show that 5 HSP90 inhibitors (AUY922, ganetespib, SNX2112, AT13387, and CUDC305) potently inhibited the proliferation of bladder cancer 5637 cells in a dose- and time-dependent manner. Our proteomic study quantified 518 twofold up-regulated and 811 twofold down-regulated proteins common to both AUY922 and ganetespib treatment. Bioinformatic analyses revealed that those differentially expressed proteins were involved in multiple cellular processes and enzyme-regulated signaling pathways, including chromatin modifications and cell death-associated pathways. Furthermore, quantitative proteome studies identified 14 types of PTMs with 93 marks on the core histones, including 34 novel histone marks of butyrylation, citrullination, 2-hydroxyisobutyrylation, methylation, *O*-GlcNAcylation, propionylation, and succinylation in AUY922- and ganetespib-treated 5637 cells. Together, this study outlines the association between proteomic changes and histone PTMs in response to HSP90 inhibitor treatment in bladder carcinoma cells, and thus intensifies the understanding of HSP90 inhibition-mediated bladder cancer therapeutics.

## Introduction

Bladder cancer is the second most common genitourinary malignancy and the fourth most commonly diagnosed cancer in males in the United States^1^. Although there has been slow yet steady progress in the development of novel chemotherapeutic strategies for the management of advanced bladder cancer in the past two decades^2–4^, current chemotherapy confers only a modest survival benefit for patients with bladder cancer, with only a small number of patients achieving long-term disease control. Health care costs, which are significant for patients diagnosed with bladder cancer, are estimated to be $4 billion per year in the United States alone. From both clinical and economic perspectives, better treatment strategies are needed for these patients.

The exploration of innovative and effective cancer treatment options has resulted in a shift in the focus of drug development from cytotoxic compounds towards targeted therapeutics that act on specific molecular targets responsible for the malignant phenotype. One promising approach is the pharmacological targeting of heat shock protein 90 (HSP90)^5–7^. HSP90 is a molecular chaperone that plays an important role in protein folding and stability of client proteins^5–7^. Thus far, over 400 HSP90 client proteins have been identified (see http://www.picard.ch/downloads/Hsp90interactors.pdf.), and these client proteins are involved in a multitude of cellular processes (*e.g*., cell cycle control and proliferative/anti-apoptotic signaling) and many are activated in malignancy^8^.

HSP90 is overexpressed in many tumors, with expression levels correlating with prognosis^9–11^. Inhibition of HSP90 function leads to the degradation of multiple oncogenic client proteins involved in tumor progression, resulting in a loss of signal transduction, growth inhibition, anti-angiogenesis, and cell death; therefore, multiple signaling pathways are simultaneously blocked by HSP90 inhibition^11, 12^. Inhibition of HSP90 has resulted in significant antitumor effects in multiple cancer animal models^13^. It has even been demonstrated as a clinical relevant biomarker in urothelial carcinoma^14^. Therefore, over 19 HSP90 inhibitors have been manufactured for the treatment of cancer in recent years, and 100 clinical trials have been undertaken to evaluate the efficacy of HSP90 inhibitors in cancer patients^15–19^. Among the HSP90 inhibitors, AUY922 (luminespib)^20, 21^, ganetespib (STA9090)^22, 23^, SNX2112^24, 25^, AT13387 (onalespib)^26^, and CUDC305^27, 28^ are novel, non-geldanamycin-derivative HSP90 inhibitors that have shown significant antitumor activity in a wide range of cancer cell lines, primary tumor cells, and animal cancer models^6, 7, 29^. Some of the preclinical activity has been observed in clinical oncology trials^29, 30^.

However, the effect and mechanism of HSP90 inhibitors in bladder carcinoma remains unclear. In the current study, we explored the efficacy of HSP90 inhibitors against bladder cancer. We demonstrate that AUY922, ganetespib, SNX2112, AT13387, and CUDC305 individually exerted a potent inhibitory effect on the growth and proliferation of human bladder cancer 5637 cells in a time- and dose-dependent manner. HSP90 inhibitors also had differential effects on cell survival and death between urothelial bladder carcinoma cells and human uroepithelial cells. Our quantitative proteomic analysis further revealed that both AUY922 and ganetespib independently induced dynamic changes in global protein expression including chromatin regulatory proteins, and that these alterations in protein levels were associated with 14 types of histone post-translational modifications (PTMs), suggesting a role for epigenetic modification in the antitumor activity of HSP90 inhibitors against bladder carcinoma. This study therefore expands our understanding of the role of HSP90 in bladder cancer and significantly furthers our mechanistic understanding of HSP90 inhibitor-mediated bladder cancer therapy.

## Results

### HSP90 inhibitors suppress cell proliferation and induce cell apoptosis in bladder cancer cells

**Figure 1:**
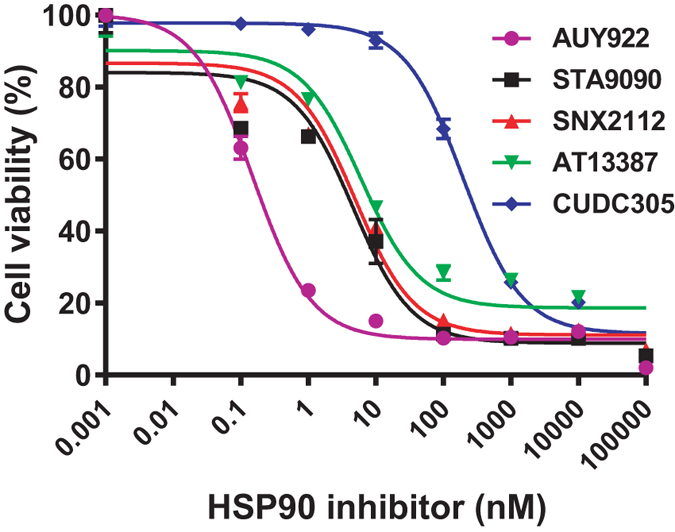
Heat shock protein 90 (HSP90) inhibitors suppress cell growth and proliferation in human bladder carcinoma cells. 5637 cells were evenly distributed in 96-well plates (5 × 10^3^ cells/well) and treated for 72 h with AUY922, ganetespib (STA9090), SNX2112, AT13387, or CUDC395 at the indicated concentrations. The ability of HSP90 inhibitors to inhibit cell growth and proliferation was determined by the MTS assay, as described in the “Methods”. Cell viability values are expressed relative to those for cells with no HSP90 inhibitor exposure (control value, 100%). The results represent the means ± SD of three independent experiments. MTS, 3-(4,5-dimethylthiazol-2-yl)-5-(3-carboxymethoxyphenyl)-2-(4-sulfophenyl)-2H-tetrazolium.

To investigate the effect of HSP90 inhibitors (AUY922, ganetespib, SNX2112, AT13387, and CUDC305) on bladder cancer cell growth and proliferation, we chose the human epithelial bladder cancer cell line 5637, which is a commonly used cell line as a model for studying bladder carcinoma. The dose-response of HSP90 inhibitor inhibition of the growth of 5637 cell line was characterized *in vitro* using the MTS assay. All of the drugs (AUY922, ganetespib, SNX2112, AT13387, or CUDC305), studied at concentrations of 0.01 nM to 100 μM, caused dose-dependent inhibition of the proliferation of 5637 cells at 24, 48, or 72 h (Table 1). As shown in Fig. 1, the half-maximal inhibitory concentration (IC_50_) values of the 5 HSP90 inhibitors at 72 h ranged 0.64 to 200 nM in 5637 cells. These results indicate that these HSP90 inhibitors potently inhibit cell proliferation and induce cell toxicity in bladder cancer 5637 cells. Similar effects of the HSP90 inhibitors were observed in several other human bladder carcinoma cell lines, including RT112, RT4, T24, T24T, FLT3, SLT3, UMUC3, UMUC5, UMUC14 (data not shown), suggesting that it is a general antitumor activity for HSP90 inhibitors in human bladder cancer cells. However, 24-h treatment did not have a dramatic effect on cell viability, suggesting that extended exposure to HSP90 inhibitors is required for them to exert their activity on cell growth and death.

**Table 1:** The half-maximal inhibitory concentration value (IC_50_) of 5 heat shock protein 90 inhibitors at different time points in bladder carcinoma 5637 cells.

Treatment	24 h*	48 h*	72 h*
AUY922	11.8	2.21	0.64
Ganetespib	106	18.5	4.28
SNX2112	109	26.3	4.77
AT13387	16,500	9.56	5.16
CUDC305	10,600	344	200

*24-h IC_50_, 48-h IC_50_, or 72-h IC_50_; Unit: nmol/L.

**Figure 2:**
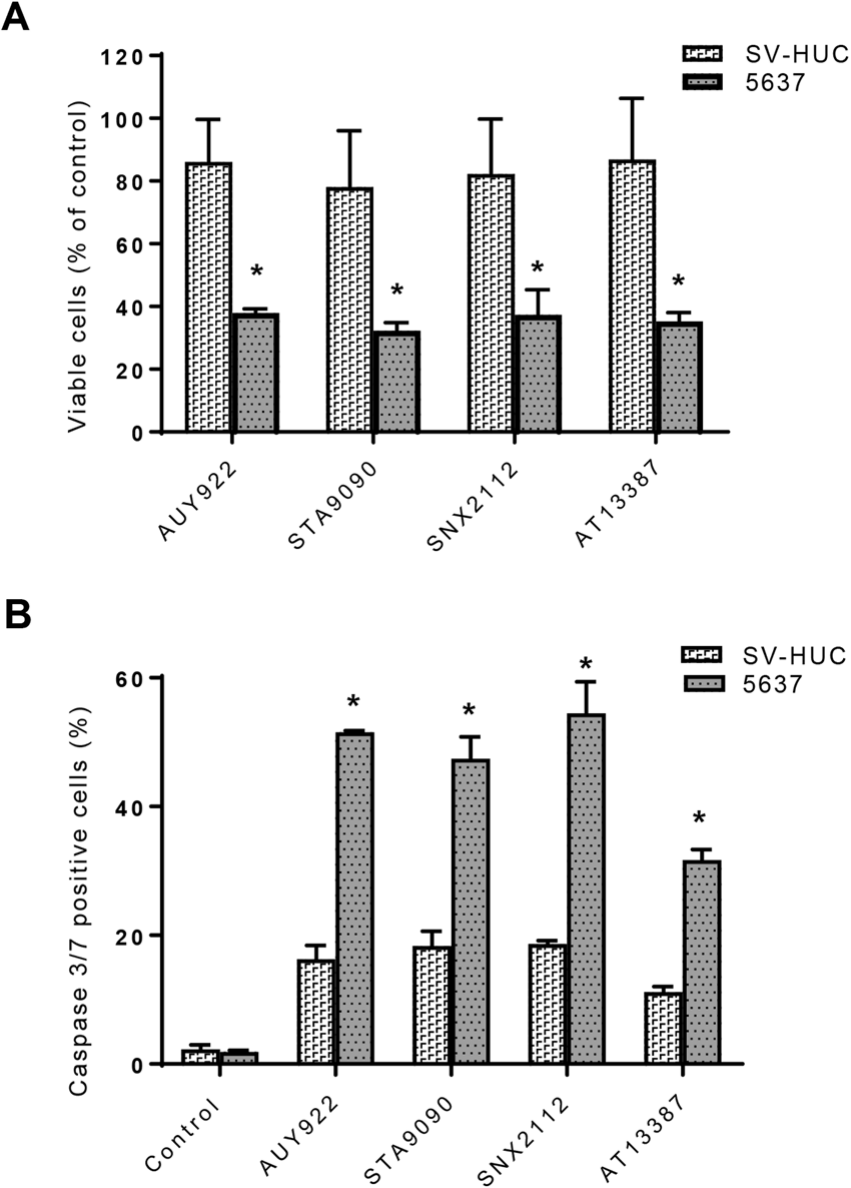
Differential effects of heat shock protein 90 (HSP90) inhibitors on cell survival and death between human uroepithelial cells and bladder cancer cells as determined by assessment of cell viability and the activity of caspases 3 and 7 using the Celigo image cytometer. 5637 and SV-HUC cells (1 × 10^4^ per well) were evenly distributed in 96-well plates overnight. Cells were incubated with AUY922 (10 nM), ganetespib (10 nM), SNX2112 (100 nM), or AT13387 (100 nM) for 48 h. (**A**) For analyzing cell viability, 5637 or SV-HUC cells were simultaneously stained with a mixture of calcein AM, propidium iodide, and Hoechst 33342 reagents for respective staining of live, dead, and all cells, and the percentage of viable cells was quantified with the Celigo imaging cytometer. Cell viability values are expressed relative to those cells without HSP90 inhibitor treatment (100% control value). *p < 0.01 versus the untreated control group; *p < 0.05 versus SV-HUC cells of the same group. (**B**) For the caspase 3/7 assay, the above mentioned HSP90 inhibitor-treated 5637 or SV-HUC cells were stained with Nexcelom ViaStain^TM^ Caspase 3/7 reagent and Hoechst 33342, as described in the “Methods”. Caspase 3/7 positive cells were identified using the Celigo imaging cytometer, and the percentage of apoptotic caspase 3/7 positive cells was calculated with the Celigo software. The data presented are representative of those obtained from three separate experiments. *p < 0.01 versus the untreated control group; *p < 0.05 versus SV-HUC cells of the same group. Control, untreated; STA9090, ganetespib.

To confirm the antitumor effect of HSP90 inhibitors in 5637 bladder cancer cells, we assessed cell viability by staining cells with live and dead cell-specific dyes using the Celigo^®^ Image Cytometry System. Treatment with AUY922, ganetespib, SNX2112, and AT13387 significantly reduced cell survival as assessed by cell viability staining. The percentages of live cells for 5637 were 37.99, 32.27, 37.32, and 35.30% in the AUY922, ganetespib, SNX2112, and AT13387 groups, respectively, as compared with the untreated control group (Fig. 2A). Similar data for the HSP90 inhibitors were obtained from UMUC1 and UMUC3 bladder carcinoma cells via assessment of cell viability (data not shown).

However, the clinical use of HSP90 inhibitors will be linked to their safety, particularly the lack of toxicity in normal cells. Therefore, we evaluated the effect of HSP90 inhibitors on cell viability in a nontumorigenic human uroepithelial cell line, SV-HUC. The cell viability data showed that the percentages of viable cells for SV-HUC were 86.04, 78.08, 82.29, and 86.87% in the AUY922, ganetespib, SNX2112, and AT13387 groups, respectively (Fig. 2A), more than twofold higher than those for the bladder cancer cell line 5637 (p < 0.05), indicating a differential effect of the HSP90 inhibitors on cell viability between the human epithelial bladder carcinoma cell line 5637 and the human uroepithelial cell line SV-HUC. Similarly, HSP90 inhibitors had differential inhibitory activity in cell growth and death between the human bladder cancer cell lines UMUC1 and UMUC3 and the human uroepithelial cell line SV-HUC (data not shown).

To further verify the differential cytotoxic activity of HSP90 inhibitor between urothelial bladder carcinoma cells and nontumorigenic human uroepithelial cells, we performed caspase 3/7 assays measuring caspases 3 and 7 activity in apoptotic cells using the Celigo image cytometer. As seen in Fig. 2B, the percentages of apoptotic caspase 3/7 positive cells for 5637 cells were 51.53, 47.42, 54.46, and 31.66% in the AUY922, ganetespib, SNX2112, and AT13387 groups, respectively, whereas the percentages of caspase 3/7 positive cells for the control SV-HUC cells were 16.29, 18.32, 18.60, and 11.17% in the AUY922, ganetespib, SNX2112, and AT13387 groups, respectively, thus showing nearly threefold differences in percentages of caspase 3/7 positive cells between the bladder cancer cells and the control cells (p < 0.05). These results indicate that HSP90 inhibitors are much more potent at inducing apoptotic cell death in human urothelial bladder cancer 5637 cells than human uroepithelial SV-HUC cells. Similar differential effect data for triggering apoptosis by the HSP90 inhibitors were also obtained between bladder carcinoma UMUC3 cells and uroepithelial SV-HUC cells (data not shown). Since the cytotoxic activity of AUY922 and ganetespib was more potent than other HSP90 inhibitors examined in 5637 cells, we chose these HSP90 inhibitors for the following proteomic experiments.

### HSP90 inhibitors stimulate dynamic changes of global protein expression in bladder carcinoma cells

To elucidate the mechanisms underlying the effect of HSP90 inhibitors on cell proliferation and cytotoxicity in bladder cancer cells, the whole cell proteome profiles of the HSP90 inhibitor-treated and -untreated 5637 cells were compared using quantitative proteomic studies. Differentially expressed proteins were identified and quantified by nanospray HPLC-MS/MS mass spectrometry. A total of 5481 non-redundant unique proteins were identified in both HSP90 inhibitor-treated and -untreated 5637 cells with 95% confidence. Of these, 4348, 4269, and 4615 were quantified in AUY922-treated, ganetespib-treated, and untreated cells, respectively. 3428 proteins were common to both HSP90 inhibitor-treated cells and -untreated cells.

Compared with the untreated control, there were 5170 differentially expressed proteins in AUY922-treated 5637 cells, including 2505 up-regulated proteins (997 ≥twofold up-regulated proteins) and 2665 down-regulated proteins (1301 ≥twofold down-regulated proteins). The fold changes ranged from 34.29 to −86.28, and 1405 of these proteins (both up- and down-regulated proteins) showed more than tenfold increased or decreased. For the ganetespib-treated 5637 cells, a total of 5187 proteins were differentially regulated; 2566 were up-regulated (1155 ≥twofold up-regulated) and 2621 down-regulated (1405 ≥twofold down-regulated). The fold changes ranged from 76.39 to −15.79, and 1524 of these proteins (both up- and down-regulated proteins) showed more than tenfold increased or decreased. 518 ≥twofold up-regulated proteins and 811 ≥twofold down-regulated proteins were common to both AUY922-treated and ganetespib-treated 5637 cells.

### Functional classification of differentially expressed proteins in bladder cancer cells following HSP90 inhibitor treatment

**Figure 3:**
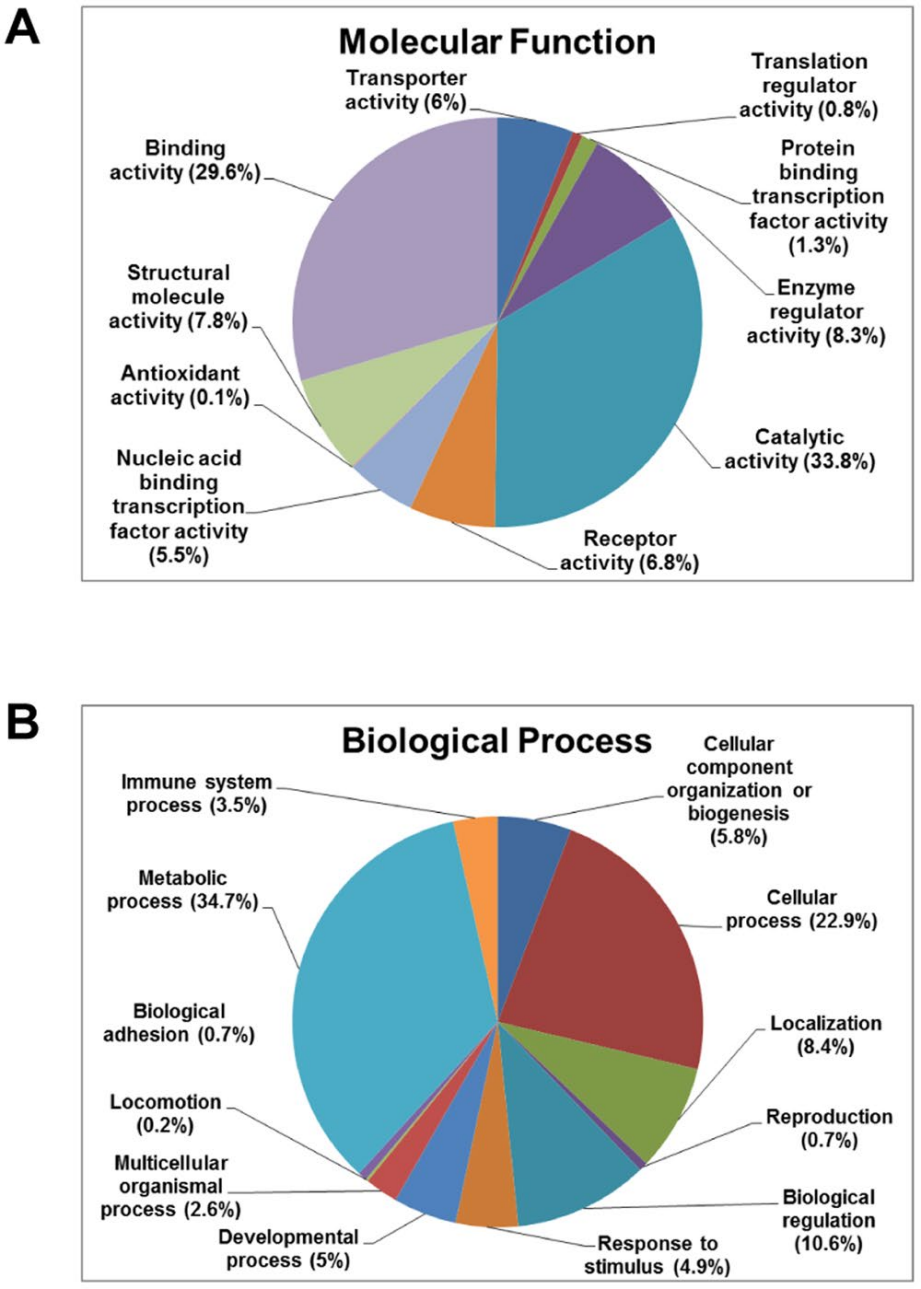
Functional categorization of the proteins that are up-regulated or down-regulated in ganetespib-treated bladder cancer cells. Differentially regulated proteins were analyzed for “functional categories” using the UnitProt knowledge database and the PANTHER classification system. Pie charts display the distribution of ≥2-fold up-regulated proteins detected in the ganetespib-treated 5637 cells based on molecular function (**A**) and ≥2-fold down-regulated proteins detected in the ganetespib-treated 5637 cells based on biological process (**B**). The percentages of the identified and quantified proteins in each category are indicated in parentheses.

To gain an initial understanding of the role and function of the identified proteins between the HSP90 inhibitor treated and untreated 5637 bladder cancer cells, we merged the protein datasets and used pathway software to provide a descriptive analysis. The functional correlation analysis of the differentially regulated proteins was done by database search using UniProt, Swiss-Prot, and the PANTHER classification systems (http://www.pantherdb.org). The categorization of differentially expressed proteins (≥2-fold up-regulated or down-regulated proteins) according to their molecular function and biological processes is shown in Fig. 3. These data are based on a compilation of proteins from the ganetespib-treated cell samples and are presented to demonstrate the range of molecular functions (Fig. 3A) and biological processes (Fig. 3B) represented by the identified proteins. Based on molecular function (Fig. 3A), the most general categories of ≥2-fold up-regulated proteins in ganetespib-treated cells were catalytic activity (33.8%), binding activity (29.6%), enzyme regulator activity (8.3%), structural molecule activity (7.8%), receptor activity (6.8%), transporter activity (6%), and nucleic acid binding transcription factor activity (5.5%). ≥Twofold down-regulated proteins in ganetespib-treated cells related to 12 biological processes (Fig. 3B), including metabolic process (34.7%), cellular process (22.9%), biological regulation (10.6%), localization (8.4%), cellular component organization or biogenesis (5.8%), developmental process (5%), response to stimulus (4.9%), and immune system process (3.5%).

A majority of the molecular functions and biological processes were affected in both AUY922-treated and ganetespib-treated bladder cancer cells. Although ganetespib caused more differentially expressed proteins (2560 ≥2-fold deregulation; 1155 ≥2-fold up-regulated and 1405 ≥2-fold down-regulated) than those caused by AUY922 (2298 ≥2-fold deregulation; 997 ≥2-fold up-regulated and 1301 ≥2-fold down-regulated proteins), the percentages of proteins in each category of the molecular function and biological process were similar between the ganetespib-treated (Fig. 3A and B) and AUY922-treated (data not shown) 5637 cells. We also compared the up-regulated proteins and the down-regulated proteins, and we showed that there were no significant differences for the percentages of proteins in each category of the molecular function and biological process between the AUY922-treated and ganetespib-treated 5637 cells (data not shown), suggesting that both AUY922 and ganetespib exert similar actions on functional categories in our cell model of bladder cancer.

### Biological pathway analysis of differentially expressed proteins using KEGG and Ingenuity pathway analysis

Next, we performed molecular pathway analysis in order to explore which cellular signaling pathways were affected by HSP90 inhibition in bladder cancer cells. We used KEGG pathway analysis to identify the biological pathways of the proteins that were significantly differentially expressed (≥twofold up-regulated or down-regulated) in the HSP90 inhibitor-treated 5637 cells. Pathway analysis using the KEGG database by the DAVID bioinformatics resources tool showed that the down-regulated proteins were associated with multiple pathways, such as oxidative phosphorylation, cell cycle, glutathione metabolism, bladder cancer, notch signaling pathway, and all major annotated DNA metabolism pathways and nucleotide related processes, including pyrimidine and purine metabolism, RNA polymerase, spliceosome, basal transcription factors, nucleotide excision repair, and DNA replication (Table 2). We also performed the same analysis for HSP90 inhibitor up-regulated proteins, which were enriched in multiple different metabolism pathways including steroid biosynthesis, N-glycan biosynthesis, valine, leucine and isoleucine biosynthesis, inositol phosphate metabolism, terpenoid backbone biosynthesis, pantothenate and CoA biosynthesis, and several protein degradation-related cellular processes, such as ubiquitin-mediated proteolysis, endocytosis, SNARE interactions in vesicular transport, adherens junctions, regulation of the actin cytoskeleton, the phosphatidylinositol signaling system, and the ErbB signaling pathway (Table 2).

**Table 2:** Main enzymatic and metabolic pathways associated with the up-regulated and down-regulated proteins in ganetespib-treated 5637 cells as analyzed by the Kyoto Encyclopedia of Genes and Genomes (KEGG).

Biological pathway	%	***P***-value
**≥Twofold up-regulated proteins**		
Steroid biosynthesis	0.7	2.3 × 10^−4^
Regulation of actin cytoskeleton	2.7	4.3 × 10^−4^
Ubiquitin mediated proteolysis	2.1	1.1 × 10^−3^
Endocytosis	2.2	1.6 × 10^−3^
Adherens junction	1.2	3.8 × 10^−3^
N-Glycan biosynthesis	0.8	1.4 × 10^−2^
SNARE interactions in vesicular transport	0.8	1.5 × 10^−2^
Valine, leucine and isoleucine biosynthesis	0.4	2.1 × 10^−2^
Phosphatidylinositol signaling system	1.0	2.3 × 10^−2^
ErbB signaling pathway	1.1	2.5 × 10^−2^
Inositol phosphate metabolism	0.8	3.1 × 10^−2^
Terpenoid backbone biosynthesis	0.4	4.9 × 10^−2^
Pantothenate and CoA biosynthesis	0.4	4.9 × 10^−2^
**≥Twofold down-regulated proteins**		
Oxidative phosphorylation	2.5	1.1 × 10^−8^
Pyrimidine metabolism	2.1	3.8 × 10^−8^
Purine metabolism	2.2	4.6 × 10^−5^
Notch signaling pathway	1.0	1.3 × 10^−4^
RNA polymerase	0.8	1.9 × 10^−4^
Spliceosome	1.8	2.7 × 10^−4^
Basal transcription factors	0.6	2.1 × 10^−2^
Pathways in cancer	2.7	2.6 × 10^−2^
Cell cycle	1.3	3.4 × 10^−2^
Glutathione metabolism	0.7	3.9 × 10^−2^
Nucleotide excision repair	0.6	4.3 × 10^−2^
Bladder cancer	0.6	4.8 × 10^−2^
DNA replication	0.6	4.9 × 10^−2^

We also analyzed the differentially expressed proteins in ganetespib-treated 5637 cells using Ingenuity Pathway Analysis (IPA) and showed that the primary pathways for the deregulated proteins (both ≥twofold up-regulated and down-regulated) are the protein ubiquitylation pathway, molecular transport, mitochondrial dysfunction, oxidative phosphorylation, 3-phosphoinositide biosynthesis, cholesterol biosynthesis, triacylglycerol biosynthesis, actin cytoskeleton signaling, notch signaling, PI3K/AKT/mTOR signaling, ErbB signaling, oxidative stress response, autophagy, nucleotide excision repair pathway, cell cycle regulation, and apoptosis signaling, which were largely consistent with the pathway analysis results using the KEGG database.

### Antitumor effect of HSP90 inhibitors in bladder cancer cells is mediated via modulation of protein expression in cell death-associated signaling pathways

Given that HSP90 inhibitors have been shown to exert a variety of anticancer activities in different types of tumors and that both AUY922 and ganetespib induced cell growth inhibition and cell death in 5637 bladder cancer cells (Figs 1 and 2 and Table 1), we explored the mechanism underlying the effect of HSP90 inhibitors on cell proliferation and cytotoxicity. We performed proteomic analyses of the HSP90 inhibitor-responsive proteome for pathways involved in cell death, and identified the differentially expressed proteins related to cell death in the cell cycle, apoptosis, oxidative stress, autophagy, and DNA damage repair pathways in response to HSP90 inhibitor treatment. Table 3 shows part of the differentially expressed proteins involved in cell death in both AUY922- and ganetespib-treated 5637 cells. These include 48 proteins involved in cell cycle progression, 23 proteins associated with the apoptosis process, 41 proteins in various DNA damage repair pathways, and 16 proteins involved in reactive oxygen species (ROS) generation and autophagy regulation. The functions and levels of the proteins in each pathway are listed in the table. Similar results were obtained in human urothelial bladder cancer UMUC3 cells treated with the HSP90 inhibitors (see Supplemental Table S1).

**Table 3:** Alterations in the levels of the proteins associated with cell death in bladder carcinoma 5637 cells in response to treatment with the heat shock protein 90 inhibitors AUY922 and ganetespib (STA9090).

Accession no.	Protein description	Symbol	Protein function	Protein level (ppm)		
				Untreated	AUY922	STA9090
**Regulation of Cell Cycle**						
5921731	G2/mitotic-specific cyclin-B2	CCNB2	Cyclin	7.83	0	0
166214910	Cyclin-C	CCNC	Cyclin	22.01	0	0
1706232	Cyclin-H	CCNH	Cyclin	28.93	0	0
218511966	Cyclin-K	CCNK	Cyclin	37.59	0	6.13
74753368	Cyclin-L1	CCNL1	Cyclin	11.84	5.99	3.78
9296942	Cyclin-T1	CCNT1	Cyclin	12.87	0	0
334302921	Cyclin-dependent kinase 1	CDK1	CDK	293.60	169.77	167.49
116051	Cyclin-dependent kinase 2	CDK2	CDK	177.66	105.75	95.39
231726	Cyclin-dependent kinase 3	CDK3	CDK	132.74	82.66	69.90
1168867	Cyclin-dependent kinase 4	CDK4	CDK	174.73	62.40	58.64
4033704	Cyclin-dependent kinase 5	CDK5	CDK	170.65	86.34	109.52
266423	Cyclin-dependent kinase 6	CDK6	CDK	152.85	77.33	119.90
68067660	Cyclin-dependent kinase 9	CDK9	CDK	167.44	59.30	85.97
6226784	Cyclin-dependent kinase 10	CDK10	CDK	25.95	0	0
34978359	Cyclin-dependent kinase 11B	CDK11B	CDK	66.60	19.82	26.82
308153421	Cyclin-dependent kinase 12	CDK12	CDK	27.17	10.58	16.69
66774048	Cyclin-dependent kinase 13	CDK13	CDK	28.84	10.42	9.40
266425	Cyclin-dependent kinase 16	CDK16	CDK	81.63	31.77	28.66
17375734	Cyclin-G-associated kinase	GAK	CDK	2.38	2.40	0
205371737	Anaphase-promoting complex subunit 4	APC4	Mitosis factor	15.42	0	0
37537861	Anaphase-promoting complex subunit 5	APC5	Mitosis factor	4.13	0	0
294862527	Anaphase-promoting complex subunit 7	APC7	Mitosis factor	20.80	0	5.93
34395509	Anaphase-promoting complex subunit 10	APC10	Mitosis factor	67.34	0	0
37537763	Cell division cycle protein 16 homolog	CDC16	Mitosis factor	20.09	0	11.46
254763423	Cell division cycle protein 23 homolog	CDC23	Mitosis factor	15.65	0	0
12644198	Cell division cycle protein 27 homolog	CDC27	Mitosis factor	7.56	0	0
52783153	Mitotic spindle assembly checkpoint protein MAD1	MD1L1	Mitosis factor	17.35	17.56	4.95
384872321	Cyclin-dependent kinase inhibitor 2A	CD2A2	CDK inhibitor	1038.10	2530.58	2611.11
19863257	Cullin-1	CUL1	Positive regulator	24.08	0	4.58
1709658	Serine/threonine-protein kinase PLK1	PLK1	Positive regulator	30.99	15.68	6.48
19858646	DNA replication licensing factor MCM5	MCM5	Positive regulator	67.89	30.05	62.93
2497824	DNA replication licensing factor MCM6	MCM6	Positive regulator	79.66	72.93	69.25
20981696	DNA replication licensing factor MCM7	MCM7	Positive regulator	99.62	78.89	84.01
76803807	Origin recognition complex subunit 1	ORC1	Positive regulator	7.23	3.66	0
8488999	Origin recognition complex subunit 2	ORC2	Positive regulator	16.19	5.46	0
8928268	Origin recognition complex subunit 3	ORC3	Positive regulator	4.38	0	4.99
6174924	Origin recognition complex subunit 5	ORC5	Positive regulator	21.48	21.73	16.34
25091097	Double-strand-break repair protein rad21 homolog	RAD21	Positive regulator	167.81	134.84	129.52
13633914	Mothers against decapentaplegic homolog 2	SMAD2	Positive regulator	13.34	6.75	5.22
51338669	Mothers against decapentaplegic homolog 3	SMAD3	Positive regulator	51.29	29.66	41.80
135674	Transforming growth factor β-1	TGFB1	Positive regulator	23.96	16.16	0
132164	Retinoblastoma-associated protein	RB	Positive regulator	3.36	0	0
20455502	Glycogen synthase kinase-3 β	GSK3B	Negative regulator	14.83	15.01	16.92
1345590	14-3-3 protein β/α	YWHAB	Negative regulator	468.41	640.51	606.65
1345593	14-3-3 protein η	YWHAH	Negative regulator	215.22	333.06	375.55
48428721	14-3-3 protein γ	YWHAG	Negative regulator	428.69	535.85	517.88
112690	14-3-3 protein θ	YWHAQ	Negative regulator	483.03	565.95	522.11
52000887	14-3-3 protein ζ/δ	YWHAZ	Negative regulator	699.13	771.74	696.15
**Regulation of Apoptosis**						
18202042	Bcl-2-like protein 11	B2L11	Pro-apoptosis	0	15.92	0
23396740	Bcl-2-like protein 13	B2L13	Pro-apoptosis	12.84	0	36.63
728945	Apoptosis regulator BAX	BAX	Pro-apoptosis	97.32	131.30	174.03
2493274	Bcl-2 homologous antagonist/killer	BAK	Pro-apoptosis	29.52	44.81	33.68
33860140	Apoptosis-stimulating of p53 protein 2	ASPP2	Pro-apoptosis	0	0	3.15
6685617	Mitogen-activated protein kinase kinase kinase 5	MAP3K5	Pro-apoptosis	0	4.59	0
13431764	Apoptosis-inducing factor 1, mitochondrial	AIFM1	Pro-apoptosis	246.76	259.10	301.42
74752283	Apoptosis-inducing factor 2, mitochondrial	AIFM2	Pro-apoptosis	8.35	33.79	28.58
125987821	Dynamin-1-like protein	DNM1L	Pro-apoptosis	21.16	0	28.97
150417955	Serine/threonine-protein phosphatase PGAM5	PGAM5	Necroptosis	560.36	665.15	725.41
12231007	Caspase-14	CASPE	Caspase	12.87	78.13	73.41
115612	Calpain small subunit 1	CPNS1	Calpain-calcium	81.34	94.07	79.55
317373596	Calpain-2 catalytic subunit	CAN2	Calpain-calcium	63.03	71.18	101.52
33112239	Calpain-7	CAN7	Calpain-calcium	0	3.88	0
57012667	Anamorsin	CPIN1	Pro-survival	9.98	0	11.38
126302556	Calpastatin	ICAL	Pro-survival	8.80	0	0
124297	Interleukin-1α	IL1A	Pro-survival	22.98	0	0
62906858	Interleukin-1β	IL1B	Pro-survival	46.31	0	0
125987833	Interleukin-1 receptor-associated kinase-like 2	IRAK2	Pro-survival	14.95	0	0
18202671	Myeloid differentiation primary response protein MyD88	MYD88	Pro-survival	10.52	0	0
21542418	Nuclear factor NF-κ-B p105 subunit	NFKB1	Pro-survival	9.65	6.51	3.67
125198	cAMP-dependent protein kinase type II-α regulatory subunit	PRKAR2A	Pro-survival	92.50	62.40	87.95
62906901	Transcription factor p65	RELA	Pro-survival	16.96	11.43	12.89
**Regulation of DNA Damage Repair**						
73921676	DNA-(apurinic or apyrimidinic site) lyase 2	APEX2	Base excision repair	12.02	0	0
37999897	Uracil-DNA glycosylase	UNG	Base excision repair	29.85	20.13	22.70
206729922	DNA-3-methyladenine glycosylase	MPG	Base excision repair	20.90	0	0
251757259	DNA ligase 3	LIG3	Base excision repair	37.04	31.23	4.22
317373290	DNA repair protein XRCC1	XRCC1	Base excision repair	49.20	0	22.45
50401132	Bifunctional polynucleotide phosphatase/kinase	PNKP	Base excision repair	23.91	12.09	27.28
17380230	Poly [ADP-ribose] polymerase 2	PARP2	Base excision repair	26.71	16.21	24.37
123369	High mobility group protein B1	HMGB1	Base excision repair	506.98	381.08	214.84
296453081	DNA repair protein complementing XP-C cells	XPC	Nucleotide excision repair	3.31	0	3.78
12643730	DNA damage-binding protein 1	DDB1	Nucleotide excision repair	188.50	77.40	99.74
12230033	DNA damage-binding protein 2	DDB2	Nucleotide excision repair	29.17	14.76	33.28
119541	TFIIH basal transcription factor complex helicase XPB subunit	ERCC3	Nucleotide excision repair	31.86	20.14	22.71
17380328	General transcription factor IIH subunit 4	GTF2H4	Nucleotide excision repair	74.15	40.92	30.76
1705722	Cyclin-dependent kinase 7	CDK7	Nucleotide excision repair	36.00	18.21	41.07
1706232	Cyclin-H	CCNH	Nucleotide excision repair	28.93	0	0
1708932	CDK-activating kinase assembly factor MAT1	MNAT1	Nucleotide excision repair	20.16	0	0
25091548	Pre-mRNA-splicing factor SYF1	XAB2	Nucleotide excision repair	87.42	51.60	45.71
108936013	Cullin-4A	CUL4A	Nucleotide excision repair	36.93	16.60	18.72
296439468	Cullin-4B	CUL4B	Nucleotide excision repair	20.47	0	7.78
1171032	DNA mismatch repair protein Msh2	MSH2	Mismatch excision repair	10.00	6.74	7.60
60392986	DNA repair protein RAD50	RAD50	Homologous recombination	71.21	60.04	67.70
17380137	Double-strand break repair protein MRE11A	MRE11A	Homologous recombination	39.59	4.45	20.07
74762960	Nibrin	NBN	Homologous recombination	41.30	8.35	18.85
116242745	DNA endonuclease RBBP8	RBBP8	Homologous recombination	3.47	3.51	0
166898077	Crossover junction endonuclease MUS81	MUS81	Homologous recombination	5.65	0	0
1705486	Bloom syndrome protein	BLM	Homologous recombination	4.40	0	5.01
38258929	DNA-dependent protein kinase catalytic subunit	PRKDC	Non-homologous end-joining	521.31	415.28	438.99
125731	X-ray repair cross-complementing protein 5	XRCC5	Non-homologous end-joining	582.87	439.11	495.12
125729	X-ray repair cross-complementing protein 6	XRCC6	Non-homologous end-joining	772.18	574.37	554.28
229462842	Chromatin assembly factor 1 subunit A	CHAF1A	Chromatin structure and modification	6.52	6.59	3.71
48428038	Aprataxin	APTX	Editing and processing nuclease	43.74	8.85	19.96
146325723	E3 ubiquitin-protein ligase SHPRH	SHPRH	Ubiquitination and modification	1.85	0	0
46577660	Ubiquitin-conjugating enzyme E2 N	UBE2N	Ubiquitination and modification	225.38	228.05	116.88
254763430	7,8-dihydro-8-oxoguanine Triphosphatase	NUDT1	Modulation of nucleotide pools	15.81	0	0
347595814	Deoxyuridine 5′-triphosphate nucleotidohydrolase, mitochondrial	DUT	Modulation of nucleotide pools	49.43	37.51	14.10
269849759	Cellular tumor antigen p53	TP53	Other related	55.47	16.03	36.16
8928568	Tumor suppressor p53-binding protein 1	TP53BP1	Other related	53.70	6.39	45.04
68565701	Telomere-associated protein RIF1	RIF1	Other related	51.65	3.82	45.99
1705919	Dual specificity protein kinase CLK2	CLK2	Other related	12.48	6.31	21.36
55976619	Pre-mRNA-processing factor 19	PRPF19	Other related	463.44	331.38	373.65
**ROS Generation**						
134665	Superoxide dismutase [Mn]	SODM	Antioxidant	939.90	865.89	640.22
311033481	Glutathione peroxidase 1	GPX1	Antioxidant	230.12	232.85	87.51
269849565	Glutathione peroxidase 8	GPX8	Antioxidant	149.01	150.77	51.00
300680960	Glutathione S-transferase θ-2	GST2	Antioxidant	12.76	12.91	0
12643338	Glutathione S-transferase κ-1	GSTK1	Antioxidant	358.28	334.64	141.50
6016173	Glutathione S-transferase ω-1	GSTO1	Antioxidant	129.22	117.68	88.46
121746	Glutathione S-transferase P	GSTP1	Antioxidant	177.96	120.04	67.68
14916998	Glutathione reductase	GSHR	Reductase	17.90	6.03	6.80
2506326	Xanthine dehydrogenase/oxidase	XDH	Oxidase	0	0	2.66
**Regulation of Autophagy**						
20178289	Interferon α21	IFNA21	Autophagy	0	0	18.80
74730233	Phosphatidylinositol 3-kinase, catalytic subunit type 3	PIK3C3	Autophagy	0	10.66	8.01
317373311	Phosphatidylinositol 3-kinase regulatory subunit β	PIK3R2	Autophagy	0	4.33	0
74762700	Phosphoinositide 3-kinase, regulatory subunit 4	PIK3R4	Autophagy	0	2.32	5.23
61212142	Autophagy-related protein 3	ATG3	Autophagy	0	10.04	0
17366828	Autophagy-related protein 5	ATG5	Autophagy	11.32	14.59	12.92
20140441	Autophagy-related protein 13	ATG13	Autophagy	0	0	6.87

ROS, reactive oxygen species.

### Heat shock protein 90 inhibitor treatment alters the levels of chromatin regulatory enzymes and proteins in bladder cancer cells

Next, we quantified dynamic change in global protein abundance of the chromatin-modifying enzymes and proteins in HSP90 inhibitor-treated 5637 bladder cancer cells. Unexpectedly, we found that the protein levels of HDAC1, HDAC2, and HDAC3 in the deacetylation complexes of Mi-2/NuRD, CoREST, NcoR, SMRT, and Sin3 were all down-regulated in both AUY922- and ganetespib-treated cells. As seen in Table 4, treatment with AUY922 and ganetespib induced 2.3-fold and 1.6-fold down-regulation for HDAC1, 2.4-fold and 1.4-fold down-regulation for HDAC2, and 3.0-fold and 2.6-fold down-regulation for HDAC3, respectively. The levels of NAD-dependent protein deacetylase sirtuin-3 were also significantly reduced in response to AUY922 or ganetespib exposure (Table 4). In contrast, the levels of the lysine acetyltransferases HAT1, KAT6A, KAT7 and CREBBP were all elevated following HSP90 inhibitor induction (Table 4). In addition, the protein levels of the lysine demethylases KDM1A, KDM2A, and KDM4B for H3K4, H3K9, and H3K36 demethylation were decreased, while the levels of the lysine demethylases KDM5A and PHF2 for H3K4 and H3K9 demethylation were increased in HSP90 inhibitor-treated cells (Table 4). Furthermore, AUY922 or ganetespib up-regulated the expression of the lysine methyltransferases EHMT2, KMT2A, SUV39H1, NSD1, and EZH2 for H3K4, H3K9, H3K27, H3K36, and H4K20 methylation, but down-regulated the expression of the lysine methyltransferases SUV420H2, SETD2, and DOT1L for H3K36, H3K79, and H4K20 methylation in 5637 cells (Table 4). Interestingly, we found that the levels of 6 chromatin-remodeling proteins in the complexes of SWI/SNF and NuRD/Mi-2 were markedly altered in cells treated with both HSP90 inhibitors (Table 4). For instance, AUY922 and ganetespib caused 2.2-fold and 1.7-fold reduction for SMARCC1, 1.6-fold and 2.3-fold reduction for ACTL6A, 3.1-fold and 1.2-fold reduction for CHD3, and 3.1-fold and 2.2-fold reduction for CHD4, respectively. Finally, the levels of 2 histone-binding proteins and 18 proteins involved in transcriptional regulation were also altered in this cell model after AUY922 or ganetespib treatment. These 18 proteins include 2 proteins involved in transcription activation, 13 proteins acting as repressors or corepressors, and 3 proteins playing dual roles as activator and repressor or coactivator and corepressor in the regulation of gene expression. Similar proteomic data were obtained in UMUC3 bladder carcinoma cells treated with the HSP90 inhibitors (see Supplemental Table S2). These results suggest that the alterations in the levels of chromatin-modifying enzymes and proteins may contribute to the altered expression of proteins, including cell death associated proteins in HSP90 inhibitor-treated bladder cancer cells.

**Table 4:** Selected differentially expressed chromatin modifying enzymes and proteins in bladder cancer 5637 cells in response to treatment with the heat shock protein 90 inhibitors AUY922 and ganetespib (STA9090).

Accession no.	Protein description	Symbol	Complex	Protein function	Protein level (ppm)		
					Untreated	AUY922	STA9090
2498443	Histone deacetylase 1	HDAC1	Mi-2/NuRD; CoREST; Sin3	Lysine deacetylase	374.75	163.45	235.90
68068066	Histone deacetylase 2	HDAC2	Mi-2/NuRD; CoREST; Sin3	Lysine deacetylase	421.20	174.35	305.81
3334210	Histone deacetylase 3	HDAC3	Mi-2/NuRD; NcoR/SMRT	Lysine deacetylase	87.32	29.45	33.21
38258651	NAD-dependent protein deacetylase sirtuin-3	SIRT3	HDAC	Lysine deacetylase	23.42	15.80	0
3334209	Histone acetyltransferase type B catalytic subunit	HAT1	KATs	Lysine acetyltransferase	52.03	67.69	67.84
215274095	Histone acetyltransferase KAT6A	KAT6A	KATs	Lysine acetyltransferase	1.55	1.57	5.32
68565854	Histone acetyltransferase KAT7	KAT7	KATs	Lysine acetyltransferase	35.68	36.10	52.34
116241283	CREB-binding protein	CREBBP	KATs	Lysine acetyltransferase	0	2.58	1.46
51315808	Lysine-specific histone demethylase 1A	KDM1A	CoREST	Lysine demethylase	32.90	22.19	20.85
38257795	Lysine-specific demethylase 2A	KDM2A	KDMs	Lysine demethylase	29.48	10.85	3.06
134047803	Lysine-specific demethylase 4B	KDM4B	KDMs	Lysine demethylase	2.84	0	0
215274124	Lysine-specific demethylase 5A	KDM5A	KDMs	Lysine demethylase	0	1.87	6.31
215274229	Lysine-specific demethylase PHF2	PHF2	ARID5B	Lysine demethylase	11.50	19.89	51.87
325511404	Histone-lysine N-methyl-transferase EHMT1	EHMT1	KMTs	Lysine methyltransferase	35.99	7.28	35.59
116241348	Histone-lysine N-methyl-transferase EHMT2	EHMT2	KMTs	Lysine methyltransferase	23.16	7.81	46.99
146345435	Histone-lysine N-methyl-transferase 2A	KMT2A	KMTs	Lysine methyltransferase	0.79	0	10.74
25091290	Histone-lysine N-methyl-transferase SUV39H1	SUV39H1	KMTs	Lysine methyltransferase	30.24	45.89	60.37
74727906	Histone-lysine N-methyl-transferase SUV420H2	SUV420H2	KMTs	Lysine methyltransferase	13.48	0	0
296452963	Histone-lysine N-methyl-transferase SETD2	SETD2	KMTs	Lysine methyltransferase	1.22	0	0
32469769	Histone-lysine N-methyl-transferase NSD1	NSD1	KMTs	Lysine methyltransferase	0	0	1.32
25090171	Histone-lysine N-methyl-transferase DOT1L	DOT1L	KMTs	Lysine methyltransferase	1.79	0	0
3334180	Histone-lysine N-methyl-transferase EZH2	EZH2	PRC2/EED-EZH2	Lysine methyltransferase	12.52	8.45	28.58
209572723	SWI/SNF complex subunit SMARCC1	SMARCC1	SWI/SNF	Chromatin remodeling	191.65	88.41	115.76
238054318	SWI/SNF-related matrix-associated actin-dependent regulator of chromatin subfamily D member 1	SMARCD1	SWI/SNF	Chromatin remodeling	120.94	79.55	103.49
322510105	SWI/SNF-related matrix-associated actin-dependent regulator of chromatin subfamily D member 2	SMARCD2	SWI/SNF	Chromatin remodeling	93.84	77.15	80.30
23396463	Actin-like protein 6A	ACTL6A	SWI/SNF	Chromatin remodeling	319.42	198.33	140.81
88911273	Chromodomain-helicase-DNA-binding protein 3	CHD3	Mi-2/NuRD	Chromatin remodeling	28.03	9.45	23.10
311033360	Chromodomain-helicase-DNA-binding protein 4	CHD4	Mi-2/NuRD	Chromatin remodeling	263.87	85.70	120.80
1172846	Histone-binding protein RBBP4	RBBP4	Mi-2/NuRD; Sin3	Histone-binding protein	300.44	163.13	200.65
2494891	Histone-binding protein RBBP7	RBBP7	Mi-2/NuRD; Sin3	Histone-binding protein	315.09	185.37	234.10
226693612	F-box-like/WD repeat-containing protein TBL1X	TBL1X	N-CoR/SMRT	Transcription activation	10.79	5.46	12.32
23396874	F-box-like/WD repeat-containing protein TBL1XR1	TBL1XR1	N-CoR/SMRT	Transcription activation	66.65	12.26	41.48
84029319	Transcriptional regulator Kaiso	ZBTB33	N-CoR/SMRT	Transcriptional regulator	9.27	4.69	0
226713806	Nuclear receptor corepressor 2	NCOR2	N-CoR/SMRT	Corepressor	6.17	0	0
6226623	G protein pathway suppressor 2	GPS2	N-CoR/SMRT	Repressor	9.52	0	0
74717977	Histone deacetylase complex subunit SAP130	SP130	Sin3	Repressor	17.83	0	3.39
68053233	Sin3 histone deacetylase corepressor complex component SDS3	SDS3	Sin3	Corepressor	37.98	0	21.67
37999759	Paired amphipathic helix protein Sin3a	SIN3A	Sin3	Repressor	88.07	17.33	86.53
68053233	Sin3 histone deacetylase corepressor complex component SDS3	SUDS3	Sin3	Repressor	37.98	0	21.67
6831678	Histone deacetylase complex subunit SAP18	SAP18	Sin3	Corepressor	81.42	82.39	69.67
212276438	Inhibitor of growth protein 1	ING1	Sin3	Tumor suppressor	14.76	0	8.42
74762776	REST corepressor 1	RCOR1	CoREST	Corepressor	25.85	0	0
6014741	C-terminal-binding protein 1	CTBP1	CoREST	Corepressor	106.17	21.49	16.15
50401198	Methyl-CpG-binding domain protein 2	MBD2	Mi-2/NuRD	Repressor	37.89	7.67	17.29
50400820	Methyl-CpG-binding domain protein 3	MBD3	Mi-2/NuRD	Repressor	10.70	0	0
259016275	Metastasis-associated protein MTA1	MTA1	Mi-2/NuRD	Coactivator and corepressor	174.23	35.26	69.57
29840793	Metastasis-associated protein MTA2	MTA2	Mi-2/NuRD	Activator and repressor	400.94	108.50	202.13
29840798	Metastasis-associated protein MTA3	MTA3	Mi-2/NuRD	Corepressor	110.10	15.92	11.96

### HSP90 inhibition modulates post-translational modifications of histones in bladder carcinoma cells

Given that HSP90 inhibitors caused alterations in the levels of histone-modifying enzymes and protein expression in bladder cancer cells, and that histone modifications play essential roles in the control and regulation of gene expression, we investigated the effect of HSP90 inhibitors on histone post-translational modifications (PTMs) in 5637 cells.

**Figure 4:**
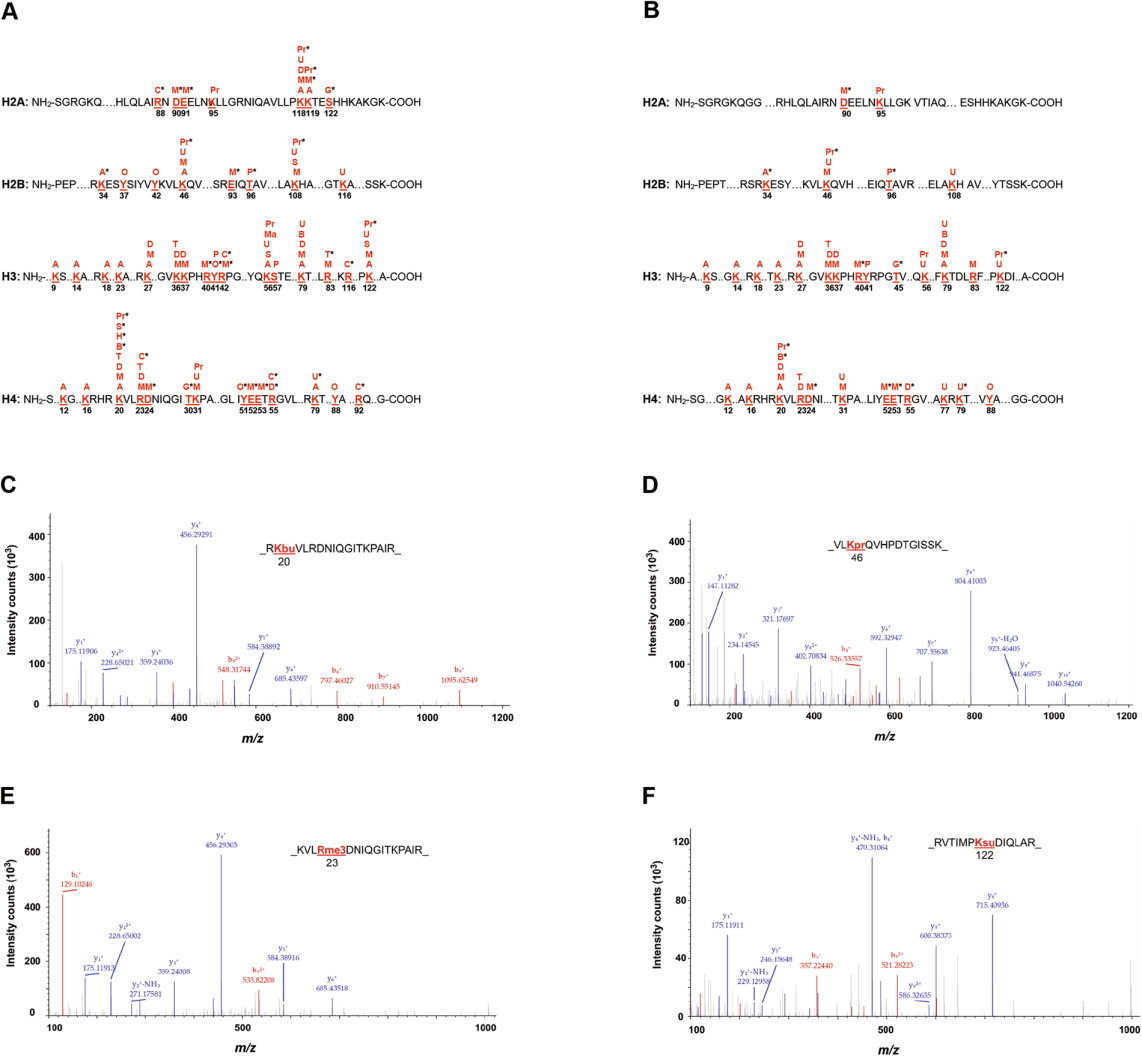
Identification of post-translational modification (PTM) residues in core histone proteins of bladder cancer cells following heat shock protein 90 inhibitor treatment. The illustration of identified PTM sites on the core histones in 5637 cells in response to AUY922 (**A**) and ganetespib (**B**) exposure. The identified PTM sites on the core histones are numbered and highlighted. *Indicates novel histone marks identified in this study. *A*, acetyl; *B*, butyryl; *C*, citrulline; *D*, dimethyl; *G*, *O*-GlcNAcyl; *H*, 2-hydroxyisobutyryl; *M*, monomethyl; *Ma*, malonyl; *O*, oxidation; *P*, phosphoryl; *Pr*, propionyl; *S*, succinyl; *T*, trimethyl; *U*, ubiquityl. (**C**) An MS/MS spectrum of a tryptic peptide histone H4K20 lysine-butyrylated peptide _RK(bu)VLRDNIQGITKPAIR. (**D**) An MS/MS spectrum of a tryptic peptide histone H2BK46 lysine-propionylated peptide _VLK(pr)QVHPDTGISSK. (**E**) An MS/MS spectrum of a tryptic peptide histone H4R23 arginine-trimethylated peptide _KVLR(me3)DNIQGITKPAIR. (**F**) An MS/MS spectrum of a tryptic peptide histone H3K122 lysine-succinylated peptide _RVTIMPK(su)DIQLAR.

To assess the impact of HSP90 inhibitors on site-specific PTMs of histones, we applied quantitative proteomics to profile histone PTMs in 5637 cells after AUY922 or ganetespib treatment, followed by protein sequence database searches for peptide identification and PTM site mapping. The diagram of Fig. 4 shows that a total of 14 different types of PTMs on the core histones were identified, including 7 recently identified histone PTM types, such as butyrylation, citrullination, *O*-GlcNAcylation, 2-hydroxyisobutyrylation, malonylation, propionylation, and succinylation. We also analyzed several other types of histone PTMs, including ADP-ribosylation, biotinylation, and crotonylation; however, we did not find these three types of histone PTMs in HSP90 inhibitor-treated 5637 cells (data not shown).

Furthermore, 47 PTM sites with 93 histone marks on the N-terminal tails of core histones were detected in AUY922- and ganetespib-treated 5637 cells, including 16 acetyl (ac) marks, 2 butyryl (bu) marks, 6 citrulline (ci) marks, 8 dimethyl (me2) marks, 1 2-hydroxyisobutyryl (hib) mark, 21 monomethyl (me1) marks, 1 malonyl (ma) mark, 3 *O*-GlcNAcyl (og) marks, 5 oxidation (ox) marks, 3 phosphoryl (ph) marks, 9 propionyl (pr) marks, 4 succinyl (su) marks, 4 trimethyl (me3) marks, and 10 ubiquityl (ub) marks (Fig. 4 and Table 5). To our knowledge, 34 of these histone marks have not been reported in any species in the past, including H2BK34ac, H4K20bu, H2AR88ci, H3R42ci, H3R116ci, H4R23ci, H4R55ci, H4R92ci, H4K20hib, H2AD90me1, H2AE91me1, H2AK119me1, H2BE93me1, H3R40me1, H3R42me1, H3R83me3, H4D24me1, H4E52me1, H4E53me1, H4R55me2, H2AS122og, H3T45og, H4T30og, H3Y41ox, H4Y51ox, H2BT96ph, H2AK118pr, H2AK119pr, H2BK46pr, H2BK108pr, H3K122pr, H4K20pr, H4K20su, and H4K79ub^31–33^.

**Table 5:** Summary of post-translational modifications (PTMs) identified on the core histones in AUY922- and ganetespib-treated bladder cancer 5637 cells.

Histone PTM type	Total histone mark	Novel histone mark
Acetylation	16	1
Butyrylation	2	1
Citrullination	6	6
2-Hydroxyisobutyrylation	1	1
Malonylation	1	0
Mono-methylation	21	9
Di-methylation	8	1
Tri-methylation	4	1
O-GlcNAcylation	3	3
Oxidation	5	2
Phosphorylation	3	1
Propionylation	9	6
Succinylation	4	1
Ubiquitylation	10	1

See Fig. 4A and B for details.

Intriguingly, we observed that PTM sites were differentially identified in core histones. For example, we only detected acetylation but not methylation on 9 histone sites (H2BK34ac, H3K9ac, H3K14ac, H3K18ac, H3K23ac, H3K56ac, H4K12ac, H4K16ac and H4K79ac), whereas only mono-methylation but not acetylation was detected on 15 histone sites (H2AD90me1, H2AE91me1, H2BK46me1, H2BE93me1, H2BK108me1, H3K36me1, H3K37me1, H3R40me1, H3R42me1, H3R83me1, H4R23me1, H4D24me1, H4K31me1, H4E52me1, and H4E53me1). Moreover, we showed that multiple PTMs were detected on single histone sites, including H2AK118, H2AK119, H2BK46, H2BK108, H3K27, H3K36, H3K37, H3Y41, H3R42, H3K56, H3K79, H3R83, H3K122, H4K20, H4R23, H4K31, H4R55, and H4K79 (Fig. 4). Representative spectra of modified histone peptides are shown in Fig. 4C–F, including the spectra for peptides of histone H4K20bu, H4R23me3, H2BK46pr, and H3K122su. In addition, some selected PTM sites identified on the core histones and the corresponding modified peptide sequences in HSP90 inhibitor-treated 5637 cells are listed in Table 6.

**Table 6:** Selected post-translational modification sites identified on the core histones and the modified peptide sequences in heat shock protein 90 inhibitor-treated bladder cancer 5637 cells.

Modified histone site	Modified peptide sequence
**Acetylation site**	
H2BK34	_KRKRSRK(ac)ESYSIY_
H3K9	_TKQTARK(ac)STGGKA_
H3K14	_RKSTGGK(ac)APRKQL_
H3K18	_GGKAPRK(ac)QLATKA_
H3K23	_RKQLATK(ac)AARKSA_
H3K27	_ATKAARK(ac)SAPATG_
H3K56	_EIRRYQK(ac)STELLI_
H3K79	_EIAQDFK(ac)TDLRFQ_
H3K122	_RVTIMPK(ac)DIQLAR_
H4K12	_GGKGLGK(ac)GGAKRH_
H4K16	_LGKGGAK(ac)RHRKVL_
H4K20	_GAKRHRK(ac)VLRDNI_
**Butyrylation site**	
H4K20	_GAKRHRK(bu)VLRDNI_
**Citrullination site**	
H4R23	_RHRKVLR(ci)DNIQGI_
H4R55	_LIYEETR(ci)GVLKVF_
**2-Hydroxyisobutyrylation site**	
H4K20	_GAKRHRK(hib)VLRDNI_
**Malonylation site**	
H3K122	_RVTIMPK(ma)DIQLAR_
**Mono-methylation site**	
H2AD90	_QLAIRND(me1)EELNKL_
H2BK108	_LPGELAK(me1)HAVSGG_
H3K27	_ATKAARK(me1)SAPATG_
H3K36	_PATGGVK(me1)KPHRYR_
H3K37	_ATGGVKK(me1)PHRYRP_
H3R40	_GVKKPHR(me1)YRPGTV_
H3R42	_KKPHRYR(me1)PGTVAL_
H3K79	_EIAQDFK(me1)TDLRFQ_
H3R83	_DFKTDLR(me1)FQSSAV_
H3K122	_RVTIMPK(me1)DIQLAR_
H4K20	_GAKRHRK(me1)VLRDNI_
H4R23	_RHRKVLR(me1)DNIQGI_
H4D24	_RHRKVLRD(me1)NIQGI_
H4K31	_NIQGITK(me1)PAIRRL_
H4E52	_ISGLIYE(me1)ETRGVL_
H4E53	_SGLIYEE(me1)TRGVLK_
H4D85	_KTVTAMD(me1)VVYALK_
**Di-methylation site**	
H3K27	_ATKAARK(me2)SAPATG_
H3K36	_PATGGVK(me2)KPHRYR_
H3K37	_ATGGVKK(me2)PHRYRP_
H3K79	_EIAQDFK(me2)TDLRFQ_
H4K20	_GAKRHRK(me2)VLRDNI_
H4R55	_LIYEETR(me2)GVLKVF_
**Tri-methylation site**	
H3K36	_PATGGVK(me3)KPHRYR_
H4K20	_GAKRHRK(me3)VLRDNI_
H4R23	_RHRKVLR(me3)DNIQGI_
***O*** **-GlcNAcylation site**	
H3T45	_HRYRPGT(og)VALREI_
**Oxidation site**	
H2BY42	_SYSIYVY(ox)KVLKQV_
H3Y41	_VKKPHRY(ox)RPGTVA_
H4Y51	_RISGLIY(ox)EETRGV_
H4Y88	_TAMDVVY(ox)ALKRQG_
**Phosphorylation site**	
H2BT96	_TSREIQT(ph)AVRLLL_
**Propionylation site**	
H2AK95	_NDEELNK(pr)LLGKVT_
H2AK118	_QAVLLPK(pr)KTESHH_
H2AK119	_AVLLPKK(pr)TESHHK_
H2BK46	_YVYKVLK(pr)QVHPDT_
H3K56	_EIRRYQK(pr)STELLI_
H3K122	_RVTIMPK(pr)DIQLAR_
H4K20	_GAKRHRK(pr)VLRDNI_
H4K31	_NIQGITK(pr)PAIRRL_
**Succinylation site**	
H3K122	_RVTIMPK(su)DIQLAR_
H4K20	_GAKRHRK(su)VLRDNI_
**Ubiquitylation site**	
H2AK118	_QAVLLPK(ub)KTESHH_
H2BK46	_YVYKVLK(ub)QVHPDT_
H2BK108	_LPGELAK(ub)HAVSGG_
H3K56	_EIRRYQK(ub)STELLI_
H3K79	_EIAQDFK(ub)TDLRFQ_
H3K122	_RVTIMPK(ub)DIQLAR_
H4K31	_NIQGITK(ub)PAIRRL_
H4K77	_TYTEHAK(ub)RKTVTA_
H4K79	_TEHAKRK(ub)TVTAMD_

**Figure 5:**
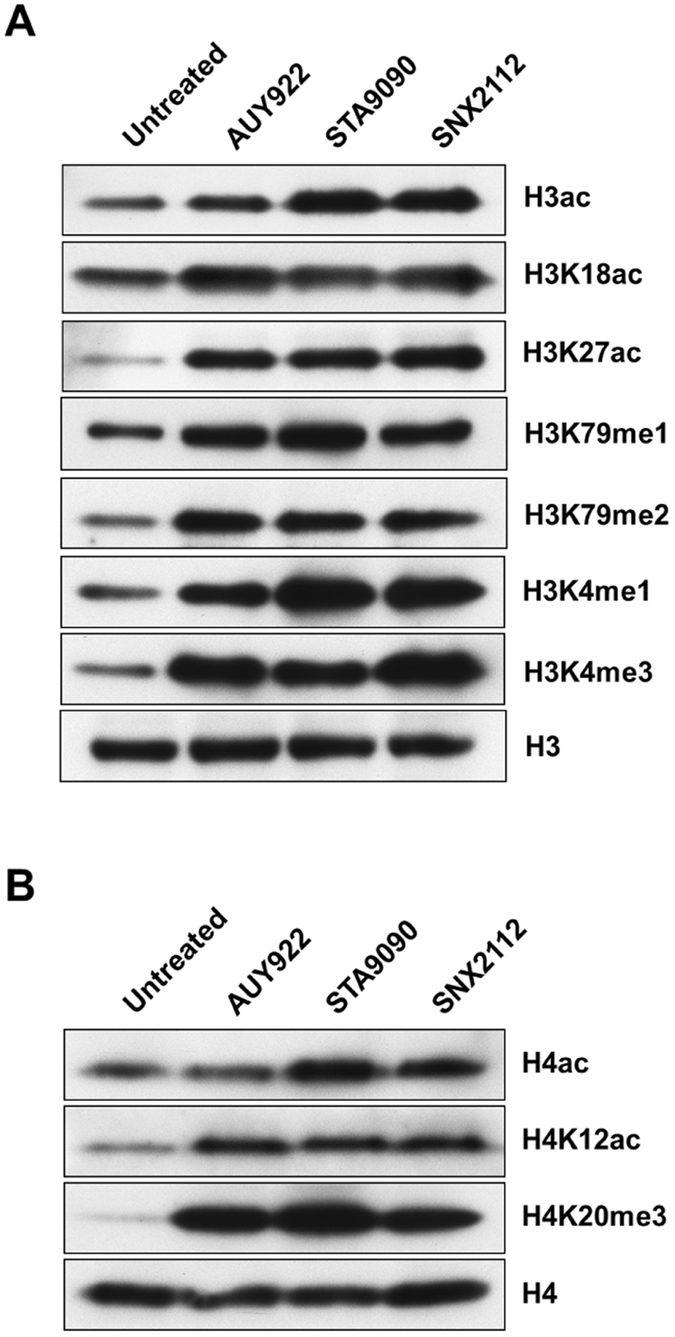
Immunoblotting for validation of HPLC-MS/MS results of histone acetylation and methylation in heat shock protein 90 inhibitor-treated bladder cancer cells. After 24 h treatment with AUY922 (100–250 nM), STA9090 (0.5–1 μM), or SNX2112 (0.5–1 μM), the 5637 cells were harvested, and whole cell protein lysates were prepared for western blot analysis of histone acetylation and methylation as described in the “Methods”. Equal protein loading was verified by using an anti-histone H3 or anti-histone H4 antibody. The levels of histone acetylation or methylation were visualized by enhanced chemiluminescence. STA9090, ganetespib.

To validate the histone PTM results from the quantitative HPLC/MS/MS analysis, we performed western blot analysis with sequence-specific antibodies to examine the dynamic change of histone modification sites upon HSP90 inhibitor treatment of 5637 cells. Consistent with the data obtained in the HPLC/MS/MS approach, our western blot data showed clear increases in the global acetyl-histone H3 and H4, as well as specific histone modifications including H3K18ac, H3K27ac, H3K79me1, H3K79me2, H4K12ac, and H4K20me3 following exposure to AUY922, ganetespib, and SNX2112 (Fig. 5). Similar results were also obtained in UMUC3 human bladder carcinoma cells induced with the same HSP90 inhibitors (data not shown). However, our experiment showed that the levels of H3K23ac were reduced in 5637 cells in response to AUY922 or ganetespib (data not shown). In addition, because some histone marks such as H3K4me and H4K8ac were not identified in HSP90 inhibitor-treated 5637 cells due to the fact that the peptide fragments containing these histone marks were too short to be detected by proteomics, we assessed the PTMs of H3K4 and H4K8 in our cell model systems by immunoblotting. Unexpectedly, we found marked increases in H3K4me1 and H3K4me3 in the three HSP90 inhibitor-treated 5637 cells (Fig. 5A). Interestingly, elevated levels of H3K4me2 and H4K8ac were observed only in HSP90 inhibitor-treated UMUC3 but not in 5637 cells (data not shown). Collectively, the profiles of histone PTMs in response to HSP90 inhibitor treatment indicate a marked impact of HSP90 inhibition on epigenetic modifications in bladder cancer cells.

## Discussion

Although HSP90 inhibitors have been evaluated on multiple regimens in different types of tumors, there is no HSP90 inhibitor currently in clinical trial for urinary bladder cancer. In the present study, we provide evidence supporting *in vitro* antitumor activity of the HSP90 inhibitors AUY922, ganetespib, SNX2112, AT13387, and CUDC305 in human bladder carcinoma cells. We also showed that HSP90 inhibitors have differential cytotoxic activity between urothelial bladder cancer cells and nontumorigenic human uroepithelial cells. Further, our quantitative proteomic analysis identified 5481 proteins, among which 518 proteins were twofold up-regulated and 811 proteins were twofold down-regulated in both AUY922- and ganetespib-treated 5637 cells. The subsequent bioinformatic analysis revealed that those quantifiable proteins were mainly involved in cellular metabolism and cell death-associated processes, including cell cycle progression, apoptotic cell death, DNA damage repair, oxidative stress, and autophagy regulation (Table 3), suggesting that those proteins in these pathways are involved in HSP90 inhibitor-induced cell death in 5637 bladder carcinoma cells.

Regulation of protein abundance in the cell is mainly through transcriptional and post-transcriptional mechanisms. Chromatin modification is one of the major epigenetic mechanisms^34, 35^, encompassing ATP-dependent chromatin remodeling and various histone modifications^36^. Chromatin modifications modulate transcription by altering the accessibility of DNA to the regulatory transcription machinery proteins, and binding of regulatory proteins (*e.g*., transcription factors or repressors) to the promoter sequence of a gene resulting in activation or blocking of transcription.

In the present study, we detected 14 different types of histone PTMs on the N-terminal tails of core histones in AUY922- and ganetespib-treated 5637 cells, suggesting a key role for HSP90 in modulating dynamics of multiple PTMs at N-terminal tails. Histone modification in the N-terminal region has been called the “histone code” or “epigenetic code”^37^, which plays a central role in chromatin remodeling and gene transcriptional regulation. Herein, 47 PTM sites with 93 histone marks were identified in HSP90 inhibitor-treated 5637 cells, and surprisingly, 34 novel histone marks detected in this study have not been reported in any species before, suggesting profound epigenetic modulation by HSP90 inhibitors in bladder cancer cells^31–33^. For instance, 16 acetyl marks on the N-terminal tails of core histones were detected in AUY922- and ganetespib-treated 5637 cells, most of which are significantly increased (Fig. 5). Histone acetylation relaxes chromatin condensation and exposes DNA to transcription factor binding, leading to an increase in gene expression^36^. Unexpectedly, we also observed that the acetylation levels of several acetyl marks were not increased (*e.g.*, H4K8ac) or even were decreased (*e.g*., H3K23ac) after HSP90 inhibitor treatment, which may impact histone code-mediated epigenetic regulation of gene expression, leading to protein level changes. Moreover, in both AUY922- and ganetespib-treated 5637 cells, we identified the methylation of H3K4, H3K36, H3K79, H3K27, and H4K20. The methylation of H3K4, H3K36, and H3K79 has been shown to be involved in transcriptional activation, whereas the methylation of H3K27 and H4K20 is involved in transcriptional repression^36^.

In addition to histone lysine acetylation and methylation, we also detected several types of less well understood histone lysine coenzyme A-dependent acylations, including butyrylation, 2-hydroxyisobutyrylation, malonylation, propionylation, and succinylation^31, 32, 38^. Similar to acetylation, these acylations neutralize the positive charge of lysine, ostensibly weakening histone-DNA contacts. Although additional work is needed to ascertain the biological relevance of this extended family of histone lysine acylations, it may be that lysine acylation is a general means to facilitate DNA access for processes such as gene transcription and DNA replication and repair.

Besides histone PTMs, dynamic remodeling of chromatin by ATP-dependent chromatin-remodeling enzymes/complexes is also involved in regulating gene expression. In the current study, we found that HSP90 inhibition caused alterations in expression of multiple proteins in the chromatin-remodeling complexes, such as SWI/SNF and NuRD/Mi-2, suggesting a role for chromatin remodeling in gene expression in HSP90 inhibitor-treated 5637 cells. It is well-documented that chromatin remodelers are involved in disassembly and reassembly of chromatin structures, leading to turning on or turning off gene transcription^39, 40^. Recent studies further suggest that chromatin-remodeling systems are universally associated with enhancers and transcriptional response elements, thereby directly participates in the transcriptional regulation of gene expression^40–43^. Although the mechanisms underlying the effect of HSP90 inhibitors on epigenetic modifications in bladder tumor cells are not understood, a growing body of evidence shows that pharmacological inhibition of HSP90 induces chromatin modifications indirectly through ubiquitin-dependent proteasomal degradation of chromatin-remodeling proteins and histone-modifying enzymes, hence altering the chromatin structure and histone code in cells^44, 45^.

**Figure 6:**
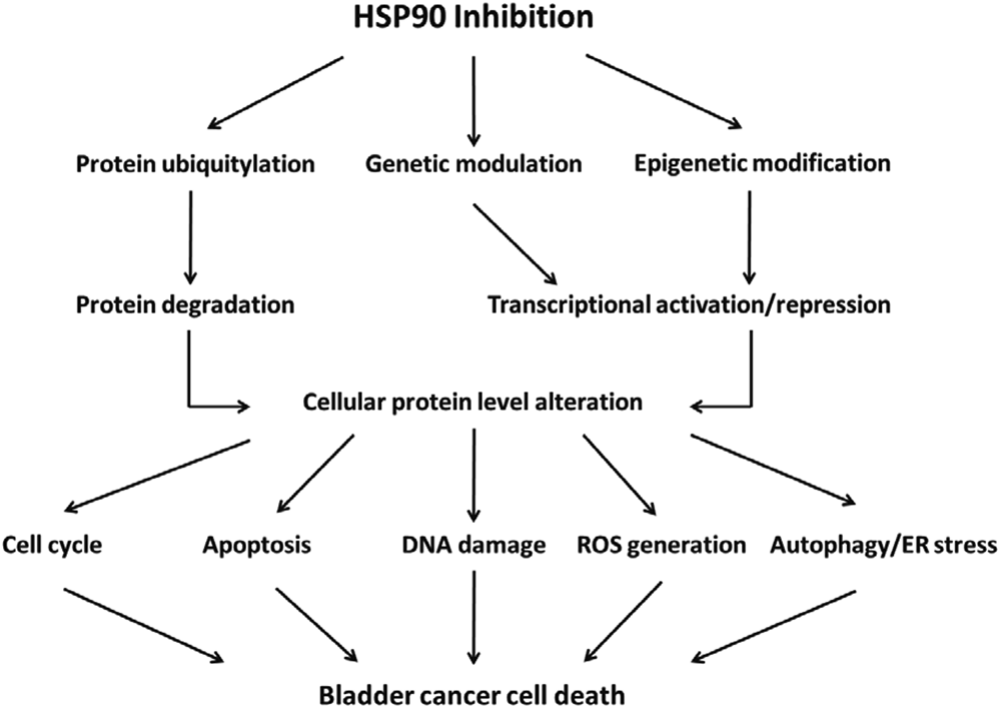
The proposed potential mechanisms of heat shock protein 90 (HSP90) inhibition lethality in bladder carcinoma cells. This schematic diagram shows that HSP90 inhibitors can substantially alter cellular protein levels directly or indirectly through inhibition of HSP90 leading to rapid ubiquitinylation and degradation of oncogenic client kinases and proteins, or through induction of genetic and epigenetic regulation of gene expression leading to changes of protein levels. These proteins with altered expression are involved in the intracellular signaling pathways of cell cycle progression, apoptotic cell death, DNA damage repair, oxidative stress, autophagy regulation, and endoplasmic reticulum (ER) stress, which are all implicated in cancer cell death. Such a mechanism may serve to integrate the roles of proteasomal degradation, genetic modulation, and epigenetic modification in culmination in misregulating cellular protein expression and cell death, which may underlie the mechanism of HSP90 inhibitor-mediated protein level alteration and cell killing in bladder cancer cells. See the text for details. ROS, reactive oxygen species.

Combining our data discussed above, we propose a possible mechanism by which HSP90 inhibitors cause bladder cancer cell growth arrest and cell death as shown in Fig. 6. In this model, HSP90 inhibitors selectively bind to HSP90, thereby inhibiting its chaperone function and promoting the degradation of oncogenic signaling proteins involved in tumor cell proliferation and survival. HSP90 inhibitors also modulate activation or repression of gene expression directly via regulation of several chromatin-remodeling proteins and histone-modifying enzymes^45–47^, or indirectly via degradation of transcriptional regulators and co-regulators, thus altering changes in the levels and activities of proteins involved in the intracellular signaling pathways of cell cycle progression, apoptotic cell death, DNA damage repair, oxidative stress, autophagy regulation, and endoplasmic reticulum (ER) stress, which are all associated with cell death. In the proposed signaling pathways depicted here (Fig. 6), AUY922 and ganetespib induce cell cycle arrest and apoptotic cancer cell death; cell cycle blockade not only causes cancer cell growth arrest, but prolonged cell cycle arrest also triggers cell suicide, usually in the form of apoptosis. In addition, the HSP90 inhibitors increase DNA damage directly or indirectly through ROS production, which in turn promotes apoptosis. On the other hand, AUY922 and ganetespib mediate cancer cell death via inducing autophagy or indirectly via ROS-mediated cell death through autophagy. Finally, HSP90 inhibition also causes accumulation of unfolded or misfolded proteins in the ER leading to ER stress and further triggering bladder carcinoma cell apoptosis^48, 49^. Because HSP90 inhibitors target cell survival and cell death through multiple closely related but distinct mechanisms, they may act collaboratively or synergistically to promote the apoptotic death of bladder cancer cells through these signaling pathways and their downstream molecular events.

In conclusion, our data demonstrate that HSP90 inhibitors exhibit potent antitumor activity against bladder carcinoma cells. Moreover, HSP90 inhibitors have differential effects on cell survival and death between human urothelial bladder cancer cells and nontumorigenic human uroepithelial cells. Proteomic data analyses further revealed alterations in protein expression involved in multiple biological functions and cell death-associated pathways in AUY922- and ganetespib-treated 5637 cells. More importantly, we found that both HSP90 inhibitors induced cytotoxicity and changes of protein expression in association with enhanced histone PTMs as well as altered levels of chromatin regulatory proteins in bladder cancer cells, reinforcing the chromatin modification activity of AUY922 and ganetespib through their HSP90 inhibition function, which subsequently impacts histone code-mediated epigenetic regulation^45–47^. Furthermore, our study identified 34 novel histone marks, and less than 5 hundred histone marks are detected within the first 50 years of histone biology (until 2015)^31, 32^. Thus, identification of the 34 new histone marks in this study is a significant advance to our understanding of “histone code”^37^. Given the known roles of histone marks in chromatin structure and function, the newly identified histone marks are likely to possess functions in transcriptional regulation and cellular metabolism. More studies may need to focus on these PTM marks in core histones, to explore the epigenetic mechanism of HSP90 inhibitor-mediated treatment of bladder tumors. Better understanding of the epigenetics underlying the HSP90 inhibition in bladder cancer may lead to the development of new treatment strategies with HSP90 inhibitors in combination with other drugs targeted at modulating relevant cell growth and death pathways^50, 51^ or at inhibiting regulatory enzymes in PTMs^52, 53^, augmenting HSP90 inhibition-mediated bladder cancer therapeutics.

## Methods

### Chemicals and reagents

The CellTiter 96 Aqueous ONE Solution Cell Proliferation Assay was purchased from Promega Corp. (Madison, WI, USA). NVP-AUY922 (AUY922) (>99% purity) was from Selleckchem.com (Houston, TX, USA). Ganetespib (STA9090) (98.79% purity) and SNX2112 (98% purity) were purchased from ApexBio (Houston, TX, USA). AT13387 (>98%) was from MedChem Express (Princeton, NJ, USA), CUDC305 (>98% purity) was from AbMole BioScience (Houston, TX, USA), and dimethyl sulfoxide (DMSO) was from Sigma-Aldrich (St. Louis, MO, USA). AUY922, ganetespib, SNX2112, AT13387, and CUDC305 were dissolved in DMSO separately and stored at −20 °C. Polyclonal antibodies against histone H3, H4, H3K4me3, H3K18ac, and H4K12ac were purchased from Abcam (Cambridge, MA, USA). Monoclonal or polyclonal antibodies against acetyl-histone H3, acetyl-histone H4, and H3K27ac were bought from EMD Millipore Corporation (Billerica, MA, USA). Monoclonal or polyclonal antibodies against histone H3K4me1, H3K4me2, H3K23ac, H3K79me1, H3K79me2, H4K8ac, and H4K20me3 were obtained from Cell Signaling Technology (Danvers, MA, USA). Restore Western Blot Stripping Buffer was from Thermo Scientific (Rockford, IL, USA). All other reagents were from Sigma-Aldrich.

### Cell culture and cell proliferation assay

The human bladder cancer cell line 5637 (HTB-9) was purchased from the American Type Culture Collection (Manassas, VA, USA). The cell line was grown in minimum essential medium (MEM), supplemented with 10% fetal bovine serum, 50 IU/ml penicillin, and 50 μg/ml streptomycin (Life Technologies; Carlsbad, CA, USA), at 37 °C in a humidified atmosphere with 5% CO_2_.

The anti-proliferative effects of AUY92, ganetespib, SNX2112, AT13387, and CUDC305 were assessed using an MTS (3-(4,5-dimethylthiazol-2-yl)-5-(3-carboxymethoxyphenyl)-2-(4-sulfophenyl)-2H-tetrazolium)-based assay (Promega), as previously described^54^. In brief, 5637 bladder carcinoma cells (5 × 10^3^ cells/well) were evenly seeded in 96-well plates with 100 µl of medium for 24 h, and then treated with AUY922, ganetespib, SNX2112, AT13387, or CUDC305 at the indicated concentrations (0, 0.01 nM, 0.1 nM, 1 nM, 10 nM, 100 nM, 1 μM, 10 μM, and 100 μM) in 100 μl of medium for 24, 48, or 72 h. At the end of incubation, 20 μl of CellTiter 96 Aqueous One Solution reagent were added to each well of the assay plates containing the treated and untreated cells in 200 μl of culture medium, and the plates were incubated at 37 °C and 5% CO_2_ for 2 h. The optical density at 490 nm was determined using a 96-well iMark^TM^ Microplate Reader (Bio-Rad Laboratories; Hercules, CA, USA). Proliferation rates were calculated from the optical densities of the HSP90 inhibitor-treated cells relative to the optical density of DMSO-treated control cells with no HSP90 inhibitor exposure (control value, 100%). The half-maximal inhibitory concentration (IC_50_) values for AUY922, ganetespib, SNX2112, AT13387, and CUDC305 at 24, 48, and 72 h in the 5637 cell line were calculated using GraphPad Prism version 6.01 (GraphPad Software; La Jolla, CA, USA) software. IC_50_ was considered as the drug concentration that decreases the cell count by 50%.

### Celigo cell survival assays

The 5637 and SV40-transformed human uroepithelial cells (SV-HUC) were trypsinized and placed in 96-well plates (Greiner Bio One, Monroe, NC, USA; Cat^#^ 655090) at a concentration of 1 × 10^4^ cells per well, and the cells were incubated overnight at 37 °C. The HSP90 inhibitors AUY922 (10 nM), ganetespib (10 nM), SNX2112 (100 nM), or AT13387 (100 nM) were added to those wells to a final volume of 200 μl per well. Cells were simultaneously stained with a mixture of calcein AM, propidium iodide, and Hoechst 33342 reagents for respective staining of live, dead, and all cells following the manufacturer’s protocol (Nexcelom Bioscience LLC, Lawrence, MA, USA; Cat^#^ CSK-V0001-1), and cell viability was quantified with Celigo^®^ S Imaging Cell Cytometer. Live and dead cell counts as well as the percentage of viable cells were calculated with the Celigo software program.

To assess the effect of HSP90 inhibitors on cell death via apoptosis, we measured the activity of caspases 3 and 7 in HSP90 inhibitor-induced apoptotic cells using the Celigo image cytometer. 5637 and SV-HUC cells were seeded overnight at 1 × 10^4^ cells per well. Cells were incubated with AUY922 (10 nM), ganetespib (10 nM), SNX2112 (100 nM), or AT13387 (100 nM) for 48 h, and then stained with Nexcelom ViaStain^TM^ Caspase 3/7 reagent (Nexcelom, Cat^#^ CSK-V0002-1) and Hoechst 33342 (Nexcelom, Cat^#^ CS1-0128), according to the manufacturer’s protocol. Staurosporine (10 μM) was used as a positive control. Apoptotic caspase 3/7 positive cells were identified by Celigo imaging cytometry, and live and dead cell counts as well as the percentage of caspase 3/7 positive cells were calculated with the Nexcelom Celigo software.

### Preparation of protein extraction, separation of proteins, and in-gel tryptic digestion

The 5637 bladder tumor cells were treated with AUY922 (100–250 nM) or ganetespib (0.5–1 μM) for 24 h, the monolayer cells were rinsed 3 times with cold PBS, and cells were then harvested. Total protein extraction from cell pellets was prepared by the following method. In brief, cell pellets were lysed in 0.4 ml lysis buffer (20 mM Tris-HCl, pH 7.5, 150 mM NaCl, 1 mM Na_2_EDTA, 1 mM EGTA, 1% Triton X-100, protease inhibitor cocktail pill). After cells were lysed, 50 μl of 10% SDS and 50 μl of 1 M DTT were added into the mixture followed by incubation at 95 °C for 10 min. The extraction was then sonicated and centrifuged at 15,000 × *g* for 10 min. Supernatants were collected and stored at −80 °C for further analysis. The protein concentration of the supernatants was determined by a BCA™ Reducing Reagent compatible assay kit (Thermo Scientific; Rockford, IL, USA).

Equal amounts of protein (130 μg) from each sample were fractioned by separation on a NuPAGE 4–12% Bis-Tris Gel (Life Technologies; Grand Island, NY, USA). Sixteen gel fractions from each lane representing one sample were treated with DTT for reduction, then iodoacetamide for alkylation, and further digested by trypsin in 25 mM NH_4_HCO_3_ solution. The digested protein was extracted, and the extracted peptides were dried and reconstituted in 20 μl of 0.1% formic acid before nanospray HPLC-MS/MS analysis was performed.

### Nanospray HPLC-MS/MS analysis

Sixteen tryptic peptide fractions from one cell sample were analyzed sequentially using a Thermo Scientific Q-Exactive hybrid Quadrupole-Orbitrap Mass Spectrometer equipped with a Thermo Dionex UltiMate 3000 RSLCnano System. Tryptic peptide samples were loaded onto a peptide trap cartridge at a flow rate of 5 μl/min. The trapped peptides were eluted onto a reversed-phase 25 cm C18 PicoFrit column (New Objective; Woburn, MA, USA) using a linear gradient of acetonitrile (3–36%) in 0.1% formic acid. The elution duration was 110 min at a flow rate of 0.3 μl/min. Eluted peptides from the PicoFrit column were ionized and sprayed into the mass spectrometer, using a Nanospray Flex Ion Source ES071 (Thermo) under the following settings: spray voltage, 1.6 kV and capillary temperature, 250 °C. The Q Exactive instrument was operated in the data dependent mode to automatically switch between full scan MS and MS/MS acquisition. Survey full scan MS spectra (*m/z* 300–2000) were acquired in the Orbitrap with 70,000 resolution (*m/z* 200) after accumulation of ions to a 3 × 10^6^ target value based on predictive AGC from the previous full scan. Dynamic exclusion was set to 20 sec. The 12 most intense multiply-charged ions (z ≥ 2) were sequentially isolated and fragmented in the Axial Higher energy Collision-induced Dissociation (HCD) cell using normalized HCD collision energy at 25% with an AGC target 1e5 and a maxima injection time of 100 ms at 17,500 resolution.

### HPLC-MS/MS data analysis

The raw MS files were analyzed using the Thermo Proteome Discoverer 1.4.1 platform (Thermo Scientific; Bremen, Germany) for peptide identification and protein assembly. For each cell sample, 16 raw MS files obtained from 16 sequential LC-MS analyses were grouped for a single database search against the Human UniProtKB/Swiss-Prot human protein sequence databases (20597 entries, 12/20/2013) based on the SEQUEST and percolator algorithms through the Proteome Discoverer 1.4.1 platform. Carbamidomethylation of cysteines was set as a fixed modification. The minimum peptide length was specified to be five amino acids. The precursor mass tolerance was set to 15 ppm, whereas fragment mass tolerance was set to 0.05 kDa. The maximum false peptide discovery rate was specified as 0.01. The resulting Proteome Discoverer Report contains all assembled proteins (a proteome profile) with peptides sequences and matched spectrum counts. Three proteome profiles were generated for the untreated control cells and two HSP90 inhibitor-treated cell samples.

### Protein quantification

Protein quantification used the normalized spectral abundance factors (NSAFs) method to calculate the protein relative abundance^55, 56^. To describe quantitatively the relative abundance, the ppm (part per million) was chosen as the unit and the 1,000,000 ppm value was assigned to each proteome profile. A ppm value at the range of 0 to 1,000,000 ppm for each identified protein in each proteome profile was calculated based on its normalized NSAF.

The ppm was calculated as follows:

$${{\rm{RC}}}_{{\rm{N}}}={10}^{6}\times {{\rm{NSAF}}}_{{\rm{N}}}$$

where RC_N_ is the relative concentration of protein N in the proteome of test sample; NSAF_N_ is the protein’s normalized spectral abundance factor; and N is the protein index.

NSAFs were calculated as follows:

$${{\rm{NSAF}}}_{{\rm{N}}}=({{\rm{S}}}_{{\rm{N}}}/{{\rm{L}}}_{{\rm{N}}})/\sum _{{i}=1}^{{n}}({{\rm{S}}}_{{i}}/{{\rm{L}}}_{{i}})$$

where N is the protein index; S_N_ is the number of peptide spectra matched to the protein; L_N_ is the length of protein N (number of amino acid residues); and *n* is the total number of proteins in the input database (proteome profile for one cell sample). The ratio of HSP90 inhibitor treated *versus* untreated control was defined as 1000 if the protein was not identified in untreated control, or as 0.001 if the protein was not identified in HSP90 inhibitor-treated sample.

### Signaling pathway analysis

Cell functions are executed and regulated by the proteome. The regulation of different cellular functions has been categorized into a number of pathways, such as cell cycle and apoptosis signaling pathways. To facilitate the proteomic analysis of the activation strength of a pathway, the pathway protein components, according to their functions, were designated as ligands, receptors, activating regulators, inhibitory regulators, or effectors, and their relative abundances (ppm) were summed. The protein list for all analyzed pathways and processes was obtained from the Kyoto Encyclopedia of Genes and Genomes (KEGG) pathway database (http://www.genome.jp/kegg/pathway.html), and their functional annotations were manually confirmed using the UniProtKB protein database and the NCBI protein database or available publications. The Ingenuity Pathway Analysis (IPA) program (http://www.ingenuity.com) was used to extract interactive networks among the proteins. A network with a score >2 was considered valid.

### Western blot analysis

The 5637 cells were exposed to AUY922 (100–250 nM), ganetespib (0.5–1 μM), or SNX2112 (0.5–1 μM) for 12–24 h. Then the cells were harvested and lysed with 200 μl of RIPA lysis buffer containing 50 mM Tris-HCl (pH 7.4), 1.0% NP-40, 0.25% Na-deoxycholate, 150 mM NaCl, 1 mM EDTA, 1 mM aprotinin, 1 μg/ml PMSF, leupeptin, and pepstatin. The protein concentration was quantified by BCA assay, and equal amounts of protein (30 μg) from the untreated control and treated cells were boiled for 5 min in Laemmli buffer, separated by 4–12% SDS-PAGE, and transferred to a polyvinylidene difluoride (PVDF) membrane (Life Technologies). The blots were probed with primary antibodies against acetyl-histone H3, H3K4me1, H3K4me2, H3K4me3, H3K18ac, H3K23ac, H3K27ac, H3K79me1, H3K79me2, acetyl-histone H4, H4K8ac, H4K12ac, and H4K20me3, followed by secondary AP-conjugated antibodies. The blots were stripped with Restore Western Blot Stripping Buffer, and reprobed with an anti-histone H3 or anti-histone H4 antibody as a loading control. The immunoreactivity was visualized by enhanced chemiluminescence (Life Technologies).

### Statistical and bioinformatic analysis

All quantitative values are presented as means ± standard deviation (SD) and compared among groups using two-way analysis of variance (ANOVA). All analyses were performed on SPSS 18.0 (SPSS, Chicago, IL, USA) for Windows. Student’s *t*-test was used to analyze the statistical significance of differences between untreated controls and HSP90 inhibitor-treated groups, or between HSP90 inhibitor-treated 5637 cells and HSP90 inhibitor-treated SV-HUC cells. All *P* values were determined using a two-sided test, and *P* < 0.05 was considered to indicate significance. Gene Ontology (GO) and Kyoto Encyclopedia of Genes and Genomes (KEGG) pathway analysis were performed using DAVID bioinformatics resources tool (DAVID v6.7, the Database for Annotation, Visualization and Integrated Discovery) with the total *Homo sapiens* genome information as the background. GO molecular function and biological process categories were analyzed separately. The adjusted *P*-value (Benjamini-Hochberg correction) cutoff is <0.05.

## Electronic supplementary material

Supplemental Table S1

Supplemental Table S2
